# Perioperative management of antiplatelet therapy in patients undergoing non-cardiac surgery following coronary stent placement: a systematic review

**DOI:** 10.1186/s13643-017-0635-z

**Published:** 2018-01-10

**Authors:** Christopher P. Childers, Melinda Maggard-Gibbons, Jesus G. Ulloa, Ian T. MacQueen, Isomi M. Miake-Lye, Roberta Shanman, Selene Mak, Jessica M. Beroes, Paul G. Shekelle

**Affiliations:** 1 Department of Surgery, David Geffen School of Medicine at UCLA, 10833 Le Conte Ave., CHS 72-247, Los Angeles, CA 90095 USA; 2 Department of Health Policy and Management, UCLA Fielding School of Public Health, Los Angeles, CA USA; 3 Veterans Affairs Greater Los Angeles Healthcare System, Los Angeles, CA USA; 4 Department of Surgery, UCSF School of Medicine, San Francisco, CA USA; 5 RAND Corporation, Santa Monica, CA USA

**Keywords:** Antiplatelet therapy, Perioperative care, Anticoagulation, Cardiology, Surgery, Bleeding, Major adverse cardiac events

## Abstract

**Background:**

The correct perioperative management of antiplatelet therapy (APT) in patients undergoing non-cardiac surgery (NCS) is often debated by clinicians. American College of Cardiology (ACC) and American Heart Association (AHA) guidelines recommend postponing elective NCS at least 3 months after stent implantation. Regardless of the timing of surgery, ACC/AHA guidelines recommend continuing at least ASA throughout the perioperative period and ideally continuing dual APT (DAPT) therapy “unless surgery demands discontinuation.” The objective of this review was to ascertain the risks and benefits of APT in the perioperative period, to assess how these risks and benefits vary by APT management, and the significance of length of time since stent implantation before operative intervention.

**Methods:**

PubMed, Web of Science, and Scopus were searched from inception through October 2017. Articles were included if patients were post PCI with stent placement (bare metal [BMS] or drug eluting [DES]), underwent elective NCS, and had rates of major adverse cardiac events (MACE) or bleeding events associated with pre and perioperative APT therapy.

**Results:**

Of 4882 screened articles, we included 16 studies in the review (1 randomized controlled trial and 15 observational studies). Studies were small (< 50: *n* = 5, 51–150: *n* = 5, >150: *n* = 6). All studies included DES with 7 of 16 also including BMS. Average time from stent to NCS was variable (< 6 months: *n* = 3, 6–12 months: *n* = 1, > 12 months: *n* = 6). At least six different APT strategies were described. Six studies further utilized bridging protocols using three different pharmacologic agents. Studies typically included multiple surgical fields with varying degrees of invasiveness. Across all APT strategies, rates of MACE/bleeding ranged from 0 to 21% and 0 to 22%. There was no visible trend in MACE/bleeding rates within a given APT strategy. Stratifying the articles by type of surgery, timing of discontinuation of APT therapy, bridging vs. no bridging, and time since stent placement did not help explain the heterogeneity.

**Conclusions:**

Evidence regarding perioperative APT management in patients with cardiac stents undergoing NCS is insufficient to guide practice. Other clinical factors may have a greater impact than perioperative APT management on MACE and bleeding events.

**Systematic review registration:**

PROSPERO CRD42016036607

**Electronic supplementary material:**

The online version of this article (10.1186/s13643-017-0635-z) contains supplementary material, which is available to authorized users.

## Background

Surgeons, cardiologists, primary care providers, and anesthesiologists frequently make decisions regarding the management of antiplatelet therapy (APT) for patients undergoing elective non-cardiac surgery (NCS). Patients with recent coronary stent implantation are challenging as clinicians balance the cardiac risks of discontinuing therapy with the bleeding risks of continuing antiplatelet agents.

Observational evidence suggests that patients with a history of percutaneous coronary intervention (PCI) are at increased risk of perioperative cardiac events. This risk is probably moderated by stent type, operative urgency, early discontinuation of APT, and time from coronary intervention [1–4]. American College of Cardiology (ACC) and American Heart Association (AHA) guidelines recommend delaying NCS until 30 days after bare metal stent (BMS) placement and ideally 6 months after drug eluting stent (DES) placement [5].

The role of APT in mitigating this risk is unclear. ACC/AHA guidelines recommend that patients receiving dual APT (DAPT, typically aspirin [ASA] and a P2Y_12_ inhibitor) undergoing elective surgery should continue ASA through the perioperative period and restart the P2Y_12_ inhibitor as soon as possible. However, the level of evidence is cited as expert opinion. Bridging therapy—the act of discontinuing oral antiplatelet agents and substituting short-acting anticoagulants or intravenous antiplatelet agents—is sometimes considered in lieu of holding APT in its entirety. Despite ACC/AHA guidelines finding no evidence to support this strategy, a 2011 survey indicated that as many as half of interventional cardiologists endorse it [6].

To guide clinicians on this daily clinical scenario, we conducted a systematic review to determine if there is sufficient literature to make evidence-based recommendations about the management of APT in patients with coronary stents undergoing elective NCS.

## Methods

This manuscript is a condensed and updated version of a report prepared for the Veterans Affairs (VA) that is free and publically available [7]. This review is reported according to PRISMA standards (Additional file 1) [8]. The key questions for this review were the following: (1) What are the risks and benefits of APT in the perioperative period after PCI? (2) Do the risks and benefits vary by timing of discontinuation and resumption of APT? and (3) Do the risks and benefits vary by type of procedure or by type of APT?

We searched PubMed, Web of Science, and Scopus for English-language articles published before October 11, 2017. The search included terms for APT, PCI, and discontinuing therapy (Additional file 2). Three key references [3, 9, 10] were used to search for additional articles. We further explored the references of included studies.

All stages of review were conducted by two or more team members working independently. After initial title and abstract screen, full-text articles presenting original data were included based on the following PICOT criteria:

1. Patients were status post PCI with stent placement, on APT, undergoing elective NCS. Studies that combined cardiac and non-cardiac surgery were excluded unless outcomes were sufficiently stratified, or the proportion of cardiac surgery was small (< 10%). Surgery was broadly defined and included major and minor procedures as well as endoscopy.
2. Interventions considered included any combination of preoperative and perioperative APT management, including bridging. Preoperative strategies included DAPT or single APT (ASA, P2Y_12_). For each preoperative strategy, multiple permutations of perioperative management were considered such as DAPT, continue both agents; DAPT, hold one agent; or DAPT, hold both agents. Bridging strategies were also considered.
3. Comparisons included alternative APT or bridging strategies, as well as stratification by time from PCI, type of surgical procedure, and by drug.
4. Outcomes included bleeding and/or major adverse cardiac events (MACE) either as a composite or independently (i.e., in-stent thrombosis rates).
5. There was no restriction on timing.

Case series with less than 10 patients and studies evaluating coronary stents not available in the USA were excluded.

Data extraction was completed in duplicate with discrepancies resolved by the entire team. The following descriptive data were extracted: sample size, mean age of patients, percent of female patients, surgical procedure category, setting, country, number of centers, stent types, preoperative and perioperative APT management, length of APT cessation, and details of bridging therapy. When preoperative and perioperative APT strategies were unclear, attempts were made to contact the corresponding authors for clarification.

The quality of randomized trials was evaluated using the Cochrane Collaboration tool for assessing risk of bias [11]. The quality of cohort studies was evaluated using items adapted from Hayden et al. [12], based on design (retrospective versus prospective), representativeness of the enrolled subjects, balancing for sampling differences, follow-up rates, and statistical methods. The overall quality of evidence was categorized based on the GRADE working group [13].

Outcomes assessed included MACE and bleeding events. We attempted to extract MACE and/or bleeding rates for each combination of pre- and perioperative APT (e.g., DAPT, hold both). MACE were typically defined as death, stent thrombosis, or myocardial infarction. If a study reported only one of these outcomes (i.e., stent thrombosis), we included it under the broader term “MACE.” Hemorrhagic outcomes were heterogeneous. We included clinically significant bleeding events as decided by consensus of surgeon members of the study team (CC, MMG, JU, IM). Definitions of bleeding for each study were extracted and are included in the data tables. Examples of clinically relevant bleeding included need for blood transfusion, re-operation, or escalation of care. Some studies used standardized criteria, such as Bleeding Academic Research Consortia (BARC) [14], but no two studies used the same criteria. Minor bleeding events (i.e., wound hematomas) were not included. We also collected the follow-up period for these outcomes.

Data were too heterogeneous for statistical pooling. Further, most studies only described one APT strategy without a comparison group. Of the studies that did compare two or more strategies, none compared the same two strategies. We were therefore unable to graph outcome rates in the traditional fashion (forest plots) but instead elected to plot MACE and bleeding outcomes stratified by pre- and perioperative APT management. This allowed visual assessment of the relationship between multiple APT strategies and event rates. Studies were further stratified across multiple variables including bridging, timing of APT discontinuation, surgical procedure (major versus minor), and duration of time between stent placement and surgery.

## Results

### Description of the studies identified by the literature search

The literature search identified 4882 possible citations; 123 articles were selected for full-text review, of which 16 studies were included in the analysis (Additional file 3). One study was a randomized trial. The remaining 15 were observational studies including two prospective cohort studies, 11 retrospective cohort studies, and two case-control studies. The included RCT did not blind participants/personnel and had unclear allocation concealment and blinding of outcome assessment (Table 1). The quality of cohort studies was variable; while most studies included a representative sample and abstracted data from medical records, methods used for assessing and adjusting differences in clinical variables were very heterogeneous (Table 2).

**Table 1:** Quality assessment for included randomized trial

Author, year	Random sequence generation	Allocation concealment	Blinding of participants and personnel	Blinding of outcome assessment	Incomplete outcome data/attrition	Selective outcome reporting	Other bias
Chu, 2016	+	?	–	?	+	+	+

Adapted from Cochrane Collaboration tool for assessing risk of bias [11]

(+) = low risk of bias; (−) = high risk of bias; (?) = unclear risk of bias

**Table 2:** Quality assessment for included observational studies

Author, year	Study design	Sample representativeness	Assessment of outcomes	Follow-up rate	Address balancing for sample differences	Statistical methods used
Alshawabkeh, 2013	Retrospective	■	■	N/A	♦	N/A
Assali, 2009	Retrospective	■	■	N/A	♦	N/A
Bolad, 2011	Retrospective	■	■	N/A	■	♦
Brotman, 2007	Retrospective	■	■	N/A	■	♦
Capodanno, 2015	Retrospective	■	■	N/A	■	■
Cerfolio, 2010	Prospective	■	■	■	■	■
Choi, 2010	Prospective	■	■	■	■	♦
Conroy, 2007	Retrospective	■	■	N/A	♦	N/A
Egholm, 2016	Case-control	■	■	N/A	■	■
Hawn, 2013	Case-Control	■	■	N/A	■	■
Marcos, 2011	Retrospective	■	■	N/A	♦	N/A
Sonobe, 2011	Retrospective	Unclear	Unclear	N/A	♦	N/A
Tanaka, 2014	Retrospective	■	■	N/A	♦	N/A
Tanaka, 2016	Retrospective	■	■	N/A	♦	N/A
Yamamoto, 2014	Retrospective	■	■	N/A	♦	N/A

Sample representativeness: all patients/consecutive sample = square

Assessment of outcomes: medical record review = square

Follow-up rate: > 80% = square

Address balancing of sample differences: yes = square, no = diamond

Statistical methods used: multivariate methods = square, univariate methods = diamond

Adapted from Hayden et al. [12]

Studies were mostly small, with five including less than 50 patients, five with 51–150 patients, and six reporting over 150 patients. Tables 3 and 4 provide full details of the included studies. The mean age was greater than 60 years old for all studies and included predominantly male patients. All studies included patients with DES, and seven of the 16 studies included patients with BMS. All of the studies were conducted at academic or Veterans Affairs (VA) medical centers, and 12 of the 16 studies were conducted at single sites.

**Table 3:** Evidence table for non-bridging studies

Author, year Study design Setting	Sample size Average age % Female	Operations included	Stent types included and average time since implantation	Antiplatelet strategy					30-day MACE rate	Bleeding Rate
				Preoperative	Perioperative			Postoperative		
Randomized trials										
Chu, 2016 [18] RCT Multi-Center Academic USA	43 procedures, 39 patients 68 years old 37%	Abdominal Other	Not specified	Dual (C/A) (32/43) Single (clopidogrel) (11/43)	Continued clopidogrel (22/43) Held all (21/43)			Not specified	Clopidogrel: 0% (0/22) Held: 0% (0/21)	Clopidogrel: 4.5% (1/22) Held: 4.8% (1/21)^1^
Observational studies										
Assali, 2009 [16] Retrospective Single Center Academic Israel	78 66 years old 20.5%	Abdominal Neuro Orthopedic Vascular Other	DES 468 days ± 223	Unclear	Dual (C/A) (17/78) Single (clopidogrel or ASA) (51/78) None (10/78)			Discretion of the surgeon	Dual: 11.8% (2/17) Single: 3.9% (2/51) None: 20% (2/10)	Dual: 17.6% (3/17) Single: 13.7% (7/51) None: 30% (3/10)^2^
Bolad, 2011 [17] Retrospective Single Center Veterans Affairs USA	220 66 years old 1.4%	Abdominal Endoscopy Ophthalmology Orthopedic Vascular	BMS, DES BMS: 22 months ± 17.3 DES: 18.2 months ± 12.6	Unclear	Dual (ASA + P2Y12) (23/220) Single (ASA) (41/220) Single (P2Y12) (5/220) None (151/220)			Not specified	Dual: 4.3% (1/23) ASA: 12.2% (5/41) P2Y12: 0% (0/5) None: 6.6% (10/151)	Not Specified
Brotman, 2007 [26] Retrospective Single Center Academic USA	114 71 years old 34.3%	Abdominal Ophthalmology Orthopedic Vascular Other	BMS, DES 236 days (125–354)	Dual (C/A) (114/114)	Continued dual (24/114) Continued ASA (2/114) Held all (88/114)			Not specified	Dual: 0% (0/24) ASA: 0% (0/2) Held: 2.3% (2/88)	Dual: 0% (0/24) ASA: 0% (0/2) Held: 1.1% (1/88)^3^
Cerfolio, 2010 [28] Prospective Single Center Academic USA	165 total 64 w/ stent For entire sample: 67 years old 25%	Other	BMS, DES Not specified	Dual (C/A) (14/33) Single (clopidogrel) (19/33) Single (“other”) (51/132) None (81/132)	Continued dual (14/33) Continued clopidogrel (19/33)			NA	4.8% (1/21)	9.5% (2/21)^4, 5^
Choi, 2010 [25] Prospective Single Center Academic Korea	27 68 years old 29.6%	Not Specified	DES 97 days ± 87	Dual (C/A) (27/27)	Held all (27/27)			Resumed when safe (median 6.5 days)	11.1% (3/27)^4^	14.8% (4/27)^4^^,6^
Egholm, 2016 [15] Retrospective Case-control Multicenter Mixed Denmark	MACE Cohort: 109 73 years old 45.5%	Endoscopy	DES Cases: 132 days (33–248) Controls: 125 days (48–224)	ASA (91%) P2Y12 (97%) DAPT—unclear	DAPT (84/109) Single (ASA) (8/109) Single (P2Y12) (13/109) None (4/109)			9% / 14% resumed ASA/P2Y12 within 7 days	None vs. DAPT: OR 3.46 (CI 0.49–24.71) SAPT vs. DAPT: OR 0.65 (CI 0.17–2.47)	NA
	Bleeding cohort: 279 71 years old 24%		DES Cases: 114 days (52–224) Controls: 166 days (70–267)	ASA (92%) P2Y12 (94%) DAPT—unclear	DAPT (217/279) Single (ASA) (24/279) Single (P2Y12) (23/279) None (15/279)			15% / 20% resumed ASA/P2Y12 within 7 days	NA	0 Events
Hawn, 2013 [1] Retrospective Case-control Subset Multicenter Veterans Affairs USA	41,989 total 568 in sub-sample For entire sample: 80.6% ≥ 60 years old 1.6% Female	Abdominal Endoscopy Neuro Ophthalmology Orthopedic Vascular Other	BMS, DES For entire sample: 5% < 6 weeks 21% 6 weeks–6 months 26% 6–12 months 48% 12–24 months	Dual (C/A) (328/568)	Continued dual (216/328) Continued ASA (36/328) Continued clopidogrel (14/328) All therapy held (62/328)			Not specified	All odds ratios not statistically different from 1.0	Not Specified
				Single (ASA) (173/568)	Continued ASA (143/173) Held ASA (30/173)					
				Single (clopidogrel) (33/568)	Continued clopidogrel (22/33) Held clopidogrel (11/33)					
				None (34/568)	None (34/34)					
Tanaka, 2014 [27] Retrospective Single Center Academic Japan	111 71 years old 13.5%	Endoscopy Other	DES 674 days ± 393	Dual (ASA + P2Y12) (93/111) Single (ASA) (12/111) Coumadin (9/111)	Held all (111/111)			Resumed when safe (mean 2 days)	Combined event rate: 2.7% (3/111)^7^	Not Specified
Yamamoto, 2014 [32] Retrospective Single Center Academic Japan	151 70 years old 18%	Abdominal Neuro Orthopedic Vascular Other	BMS, DES Not specified	Dual (ASA + P2Y12) (114/151)	Continued dual (63/151)	“Dual APT Group” (63/151)		Not specified	0%^4^	Dual APT group: 9.5% (6/63) Single APT -Heparin: 2.9% (2/68) Single APT + Heparin: 0% (0/20)^4,8^
					Held P2Y12 (51/151)	“Single APT Group” (88/151)	(+) Heparin (20/88)			
				Single (ASA) (37/151)	Continued ASA (37/151)		(−) Heparin (68/88)			

*APT* antiplatelet therapy, *ASA* aspirin, *BMS* bare metal stent, *C/A* clopidogrel/aspirin, *DES* drug eluting stent, *MACE* major adverse cardiac event, *NA* not applicable, *Neuro* neurosurgical procedures, *OR* odds ratio, *RCT* randomized controlled trial

^1^Bleeding-related rehospitalization, reoperation, transfusion, or mortality

^2^Hb drop > 2 g/dL

^3^Bleeding requiring return to OR or bleeding in a critical location (intracranial, retroperitoneal)

^4^Follow-up period not specified

^5^Bleeding requiring return to OR

^6^Bleeding requiring transfusion or return to OR

^7^Follow-up period ≤ 7 days

^8^Bleeding requiring transfusion, intracranial bleeding, Hb drop > 5 g/dL, and bleeding causing death within 7 days

**Table 4:** Evidence Table for Bridging Studies

Author, year Study design Setting	Sample size Average age % Female	Operations included	Stent types included and average time since implantation	Antiplatelet strategy				30-day MACE rate	Bleeding Rate
				Preoperative	Perioperative	Bridge	Postoperative		
Alshawabkeh, 2013 [19] Retrospective Single center Academic USA	51 65 years old 0%	Abdominal Endoscopy Neuro Orthopedic Vascular Other	DES 13.9 months ± 1.7	Dual (C/A) (51/51)	Held all (18/51) Held clopidogrel (33/51)	Admit day after clopidogrel discontinued, IIb/IIIa drip initiated	Clopidogrel resumed at discretion of surgeon (mean 1.2 days)	7.8% (4/51)^1^	7.8% (4/51)^1,2^
Capodanno, 2015 [22] Retrospective Multi-center Academic Italy	515 68 years old 21%	Abdominal Endoscopy Neuro Ophthalmology Orthopedic Vascular Other	BMS, DES 30% < 180 days	Dual (C/A) (162/515) Single (ASA) (353/515)	Continued dual (108/515) Continued ASA (158/515) Held all (251/515) -->LMWH	Clopidogrel held at least 5 days, ASA held at least 2 days prior to procedure; LMWH bridge	LMWH continued until APT resumed (timing not specified)	LMWH: 7.8% (14/179) No LMWH: 0.6% (1/179)	LMWH: 22.3% (40/179) No LMWH: 13.4% (24/179)^3^
Conroy, 2007 [20] Retrospective Single center Academic Australia	22 pts., 42 procedures Not specified	Abdominal Endoscopy Other	DES Not specified	Dual (C/A) (39/42)	Continued dual (21/39) Held clopidogrel (18/39)	LMWH bridge (2/18) IIb/IIIa bridge (2/18) No bridge (14/18)	Not specified	Dual: 0% (0/21) Dual held, +bridge: 0% (0/4) Dual held, −bridge: 21% (3/14)^1^	Dual: 4.8% (1/21) Dual, held, ±bridge: 0% (0/18)^1,4^
Marcos, 2011 [21] Retrospective Single center Academic Netherlands	36, 21 non-cardiac For entire sample: 66 years old 31%	Abdominal Endoscopy Orthopedic Other	DES For entire sample: 80 days ± 66	Dual (C/A) (36/36)	Held all (7/36) Held clopidogrel (29/36)	Discontinued clopidogrel 5 days preop, admit 3 days prior, tirofiban drip Discontinued clopidogrel 5 days preop, admit 2 days prior, tirofiban drip	If no risk of bleeding, clopidogrel restarted 12-24 h postop If high-risk, heparin drip until risk was lower	0%	19% (4/21)^5^
Sonobe, 2011 [23] Retrospective Single center Academic Japan	38 71 years old 13%	Other: Major Thoracic	BMS, DES 31 months	Dual (ASA + P2Y12) (16/38) Single (ASA) (21/38) None (1/38) Coumadin (9/38)	Held all (38/38)	(+) Heparin drip (16/38) (−) Heparin drip (22/38)	At discretion of surgeon (median 4 days)	(+) Heparin: 0% (0/16) (−) Heparin: 0% (0/22)	(+) Heparin: 0% (0/16) (−) Heparin: 0% (0/22)
Tanaka, 2016 [24] Retrospective Single center Academic Japan	210 71 years old 15.7%	Abdominal Endoscopy Ophthalmology Orthopedic Vascular Other	DES 31.9 months ± 23	Dual (ASA + P2Y12) (129/210) Single (ASA) (60/210) Single (P2Y12) (19/210) Coumadin (20/210)	Held all (210/210)	Heparin drip	Restarted heparin drip 2–6 h (low risk) or 10–12 h (high risk) after surgery; APT restarted mean 4.5 days after surgery	0% (0/210)	7.6% (16/210)^6^

*APT* antiplatelet therapy, *ASA* aspirin, *BMS* bare metal stent, *C/A* clopidogrel/aspirin, *DES* drug eluting stent, *LMWH* low molecular weight heparin, *MACE* major adverse cardiac event,*Neuro* neurosurgical procedures

^1^Follow-up period not specified

^2^GUSTO criteria, moderate/severe [33]

^3^Bleeding Academic Research Consortium (BARC) ≥ 2 [14]

^4^Bleeding complication, such as reoperation

^5^Bleeding requiring transfusion or reoperation

^6^TIMI major/minor [34]

Within the study by Hawn et al., there were two distinct analyses relevant to our question. The first was a retrospective cohort of 41,989 VA patients who underwent NCS within 24 months of stent placement. The second was a case-control design of 284 patients with confirmed MACE, comparing to controls without MACE, looking specifically at APT management. A second case-control study was included that evaluated MACE and bleeding events in patients undergoing gastroscopy following DES placement. This study utilized two nested-case controls to evaluate cases (bleeding, MACE) compared to matched controls, focusing on the effect of APT management [15].

### Antiplatelet and bridging strategies

Each study included one or more APT strategy, with or without bridging. For two studies [16, 17], we were unable to determine preoperative APT. Details of these two are included in the tables but are not in the figures or our analysis. Similarly, the case-control studies did not have event rates [1, 15]. We therefore had 12 studies with both pre and perioperative APT strategies with sufficient data to calculate outcome rates. Because studies could describe more than one strategy, there was a total of 17 MACE data points and 17 bleeding data points. Preoperative APT management included DAPT (usually clopidogrel and ASA), single APT (SAPT, usually ASA), or no APT. Five of the 10 studies grouped patients on preoperative DAPT and SAPT together. For each preoperative APT, there were multiple permutations of continuing or holding one or more therapies in the perioperative period. Further, six of the 12 studies also included bridging strategies. In sum, we describe six pre-perioperative APT strategies: DAPT, continue both (*n* = 3); DAPT, continue one (*n* = 1); DAPT, stop both (*n* = 2); DAPT or SAPT, stop all (*n* = 2); DAPT or SAPT, continue all (*n* = 2), DAPT or SAPT, continue clopidogrel only (*n* = 1). Bridging studies (*n* = 6) are discussed separately in this review.

### Outcomes from a randomized trial

We identified one RCT that met most of our inclusion criteria [18]. This study of NCS in a USA academic setting randomized patients to continue or stop perioperative clopidogrel. Aiming for an enrollment of 3142 patients, the studied approached 4000 individuals. Only 48 were eligible and randomized, with 39 patients successfully completing the protocol undergoing 43 procedures. Only 72% of patients completing the study were post-PCI with stent placement. No data were available on type of stent or time since deployment. Seventy-four percent of patients were on DAPT preoperatively compared to 26% on clopidogrel only. There were no MACE in either group, and there was one bleeding-related re-hospitalization in each group with a follow-up of 90 days.

### Outcomes related to antiplatelet strategy

**Fig. 1:**
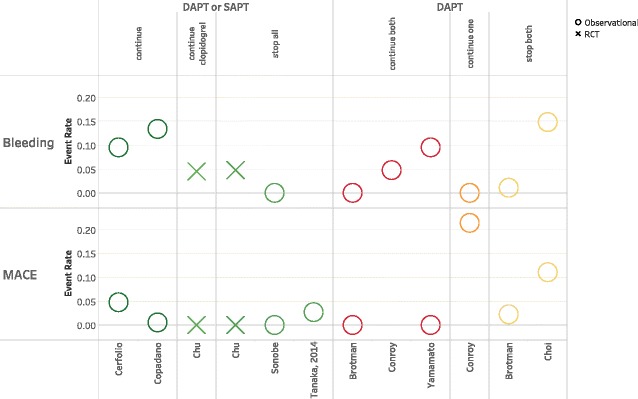
Bleeding and MACE event rates by antiplatelet strategy, including study design

Given the limitations of the one RCT, we broadened our review to include available observational evidence. MACE and bleeding rates ranged from 0 to 21% and 0 to 22% across studies, regardless of APT or bridging strategy. Among non-bridging studies, there was no association between APT strategy and outcome (Fig. 1). For example, four studies reported 0% MACE rates across three different APT strategies. Further, among the studies that used DAPT preoperatively, the study with the highest MACE event rate (21.4%) continued SAPT whereas the studies that stopped both agents had less than half the MACE rates (11.1% and 2.3%). For bleeding, three studies reported 0% rates representing three different APT strategies. The highest rate (14.8%) was reported in a study where both agents were stopped perioperatively. The case-control component of Hawn et al. found no difference in the odds of MACE across nine different APT strategies. The gastroscopy case-control study found higher odds of MACE in patients who held all APT therapy compared to patients who continued DAPT, however, this finding was not statistically significant (OR 3.46, CI 0.49–24.71). Further, the gastroscopy case-control study focused on bleeding found no re-bleeding events, and therefore no association with APT strategy.

### Perioperative bridging strategies

**Fig. 2:**
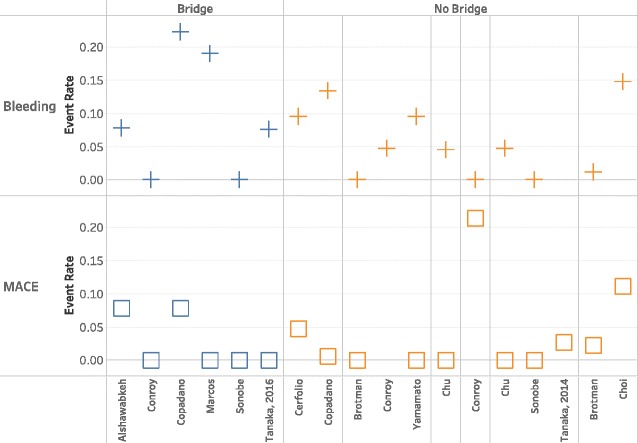
Event rate stratified by antiplatelet strategy and bridging versus no bridging

Six of the studies described perioperative bridging strategies. No two studies used the same strategy. Bridging agents included glycoprotein IIb/IIIa inhibitors [19–21], low molecular weight heparin [20, 22], and unfractionated heparin [23, 24]. APT management varied within the bridging studies: some included just DAPT while others included DAPT or SAPT. Studies also varied in perioperative management of APT with some holding only the P2Y_12_ inhibitor, while others held both the P2Y_12_ inhibitor and ASA. Further, timing of discontinuation of APT, length of bridge, and need for preoperative hospitalization were different between these studies. For the bridging studies, MACE rates ranged from 0 to 7.8% and bleeding from 0 to 22% (Fig. 2).

### Timing of discontinuation of antiplatelet agents

The timing of discontinuation of APT cessation varied between studies and among individual patients within studies. Most studies in which APT was discontinued preoperatively reported either a median or range of days of preoperative discontinuation. For the majority of these studies, APT agents were discontinued between 3 and 10 days preoperatively. No study systematically assessed the impact of timing of APT cessation on clinical outcomes. Postoperative management of APT was either not described, highly heterogeneous, or up to the discretion of the provider (Tables 3 and 4).

### Time between stent implantation and surgery

**Fig. 3:**
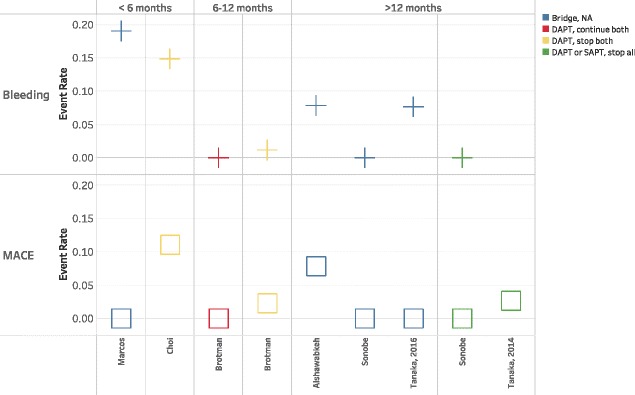
Event rate stratified by antiplatelet strategy and time since PCI

Ten studies provided a measure of average time from PCI to NCS. Three studies averaged less than 6 months (“early”) [15, 21, 25], one was six to 12 months (“mid”) [26], and six were greater than 12 months (“late”) [16, 17, 19, 23, 24, 27]. Seven of these studies had outcomes associated with pre and perioperative APT strategies providing nine MACE data points and eight bleeding data points (Fig. 3). Measures of dispersion (standard deviations, interquartile regions) were large, such that a significant fraction of the cases may have crossed time categories. Further, APT management strategies and the use of bridging varied within each time category. MACE rates for the early group were 0–11%, for the mid group 0–2.3%, and the late group 0–7.8% (Fig. 3). The retrospective cohort component of Hawn et al. found that the risk of MACE declined with increasing time since stent placement, with a rate of 11.6% for those < 6 weeks, 6.4% for those 6 weeks to 6 months, 4.2% for 6 months to 1 year, and 3.5% for 1 year to 2 years.

### Type of surgery

Most studies included a mix of surgical procedures, both major and minor, across surgical disciplines. Studies did not provide enough detail to assess outcomes for a given surgical specialty, let alone individual procedures. Two studies [23, 28] addressed only thoracic surgery. However, the studies had small samples (21 and 38 patients) and different APT management strategies. One included bridging and the other did not. MACE rates were 0 and 5%, and bleeding rates were 0 and 9.5%. The retrospective cohort component of Hawn et al. identified type of surgery as a predictor of MACE, with respiratory, vascular, and digestive operations carrying significantly higher risk (10.6, 8.4, and 8.1%, respectively) than eye/ear and integumentary operations (1.7 and 3.0%, respectively).

### Grading the quality of evidence

We judged the overall quality of evidence as very low, based on serious limitations in study design, consistency of results, and precision of the estimates.

## Discussion

The principle conclusion from our systematic review is that the available literature is insufficient to guide perioperative APT management in patients with coronary stents undergoing NCS. Further, the results suggest that clinical factors other than APT management may be more responsible for MACE and bleeding rates such as indication and urgency of operation, timing since stent placement, invasiveness of the procedure, preoperative cardiac optimization, and functional status.

Our results do not imply that APT management is inconsequential. There are a number of reasons to believe that APT management has a causal role in MACE and bleeding rates. Aspirin selectively inhibits cyclooxygenase (COX)-1 resulting in irreversible inhibition of the platelet activator thromboxane A2, while P2Y_12_ inhibitors (clopidogrel, prasurgrel, ticagrelor) inhibit P2Y_12_ ADP receptors—both mechanisms ultimately reducing platelet activation [29]. However, current available evidence is insufficient to conclude these physiologic rationales translate into appreciable differences in MACE and bleeding outcome rates dependent on perioperative APT management strategies.

The 2014 guidelines from the AHA/ACA recommended delaying elective NCS for 1 year after DES and 30 days after BMS placement. In patients undergoing urgent NCS, they recommended continuing DAPT therapy for 4–6 weeks unless the risks of bleeding outweigh the risks of stent thrombosis [30]. The 2016 update modified the former of these recommendations by reducing the window from 1 year to 6 months and also considering operations after 3 months if the risk of delaying surgery is greater than the risk of stent thrombosis [5]. These recommendations were based on contemporary evidence suggesting reduced risk of stent thrombosis with newer generation DES and on the study from Hawn et al.

The two components of Hawn et al. included a retrospective review of over 40,000 patients undergoing NCS after stent implantation as well as a case-control subset of cases (those with MACE) versus controls to ascertain the role of APT strategies in MACE outcomes. Multivariate analysis of the first component showed that non-elective surgery, recent myocardial infarction, elevated cardiac risk index, the presence of heart failure, age, the presence of chronic kidney disease, and type of operation were all associated with increased MACE risk. They further showed a sharp decline in MACE rates from time since stent implantation with an asymptote at approximately 6 months. Their case control subset looked at nine different APT strategies with no difference in the odds of MACE. The 2016 update to ACC/AHA guidelines repeat the 2014 recommendations regarding APT management encouraging providers to continue ASA through the perioperative period, and if previously on DAPT, to restart the P2Y_12_ inhibitor shortly after surgery. The result of the studies included in our review, and specifically that of Hawn et al., suggest that the importance of APT agent is likely small compared to other clinical factors.

This systematic review is limited primarily by the quality and quantity of the available evidence. Only one RCT evaluated the management of APT in patients undergoing NCS, but this study was very small (< 40 patients) and included almost 30% of patients without a history of coronary stent (not our main population of interest). Observational studies in this space are insufficient to make up for the paucity of randomized evidence. Many of the included observational studies lacked a control group rendering comparisons impossible. The few studies that did include a control group did not address sample imbalances. Case control studies are not directly comparable to cohort studies as they do not provide actual event rates. Further, patients within and across studies were heterogeneous along multiple domains including time since stent implantation, indication for procedure, and type of surgery. APT management within a given study was similarly heterogeneous using multiple APT strategies, inconsistent timing of discontinuation and restarting therapy, as well as the use of bridging.

Given the likelihood of confounding, observational studies will have difficulty in identifying or balancing these factors rendering further observational studies of the types identified in this review of little utility. Randomized studies would address this concern but inherently struggle with other limitations—namely sample size. The one RCT addressing our question enrolled only 39 patients. To detect a reduction in MACE from 5% to 3% would require a sample of approximately 1500 patients in each arm. Any further stratification, such as looking at different types of surgical procedure, or including more than one APT strategy, would require increasing this sample size further. While challenging, a sufficiently powered RCT should remain the goal. RCTs evaluating patients with cardiac disease are often capable of enrolling many thousands of patients suggesting that a study of perioperative APT management is feasible.

## Conclusions

Published studies of the association between perioperative APT management and outcomes in patients with coronary stents undergoing NCS have challenging methodologic limitations and heterogeneous results. The results suggest that clinical factors other than perioperative APT management may be more responsible for MACE and bleeding outcomes. A large clinical trial or exceptional observational study will be needed to definitively provide evidence about this clinical decision. We conclude that there is insufficient evidence to guide perioperative APT management in patients with coronary stents undergoing NCS. For now, the decision must be tailored to the situation, guided by a collaborative approach between the surgeon, cardiologist, anesthesiologist, and the patient.

## Additional files

Additional file 1: PRISMA Checklist. (DOCX 23 kb)

Additional file 2: Search terms. (DOCX 13 kb)

Additional file 3: PRISMA Flow Diagram. (PDF 91 kb)

## Abbreviations

ACC/AHA: American College of Cardiology / American Heart Association
APT: Antiplatelet therapy
ASA: Aspirin
BMS: Bare metal stent
DAPT: Dual antiplatelet therapy
DES: Drug eluting stent
MACE: Major adverse cardiac event
NCS: Non-cardiac surgery
PCI: Percutaneous coronary intervention
SAPT: Single antiplatelet therapy
VA: Veterans Affairs
