# Supplementary material for: Perioperative management of antiplatelet therapy in patients undergoing non-cardiac surgery following coronary stent placement: a systematic review
Source: Syst Rev. 2018 Jan 10;7:4. doi: 10.1186/s13643-017-0635-z (PMC5763575; doi:10.1186/s13643-017-0635-z)
Supplement: Supplementary file 2 — Search terms. (DOCX 13 kb) [file 13643_2017_635_MOESM2_ESM.docx]

**Additional file 2: Search Terms**

DATABASE SEARCHED & TIME PERIOD COVERED: PubMed – From inception to 10/11/2017

LANGUAGE: English

SEARCH STRATEGY:

"Platelet Aggregation Inhibitors"[Mesh] OR antiplatelet therap* OR anti-platelet therap*

AND

pci OR percutaneous coronary intervention*

NOT

stop OR stopped OR stopping OR discontinu*

=====================================================================

DATABASE SEARCHED & TIME PERIOD COVERED: PubMed – From inception to 10/11/2017

LANGUAGE: English

SEARCH STRATEGY:

“Similar Article” searches on the following articles:

van Kuijk, J. P., W. J. Flu, et al. (2009). "Timing of noncardiac surgery after coronary artery stenting with bare metal or drug-eluting stents." Am J Cardiol 104(9): 1229-1234.

Sandeep Singla, Sandeep, Rajesh Sachdeva, , Barry F. Uretsky, (2012). “The Risk of Adverse Cardiac and Bleeding Events Following Noncardiac Surgery Relative to Antiplatelet therapy in Patients With Prior Percutaneous Coronary Intervention.” Journal of the American College of Cardiology Vol. 60, No. 20, 2005-16.

Albaladejo, P., E. Marret, et al. (2011). "Non-cardiac surgery in patients with coronary stents: the RECO study." Heart 97(19): 1566-1572.

=====================================================================

DATABASES SEARCHED & TIME PERIOD COVERED: Web of Science and Scopus – From inception to 10/11/2017

LANGUAGE: English

SEARCH STRATEGY: “Forward (Citation)” searches on the 3 articles cited above.
