# Supplementary material for: Perioperative management of antiplatelet therapy in patients undergoing non-cardiac surgery following coronary stent placement: a systematic review
Source: Syst Rev. 2018 Jan 10;7:4. doi: 10.1186/s13643-017-0635-z (PMC5763575; doi:10.1186/s13643-017-0635-z)
Supplement: Supplementary file 3 — PRISMA Flow Diagram. (PDF 91 kb) [file 13643_2017_635_MOESM3_ESM.pdf]

Additional File 3: PRISMA Flow Diagram

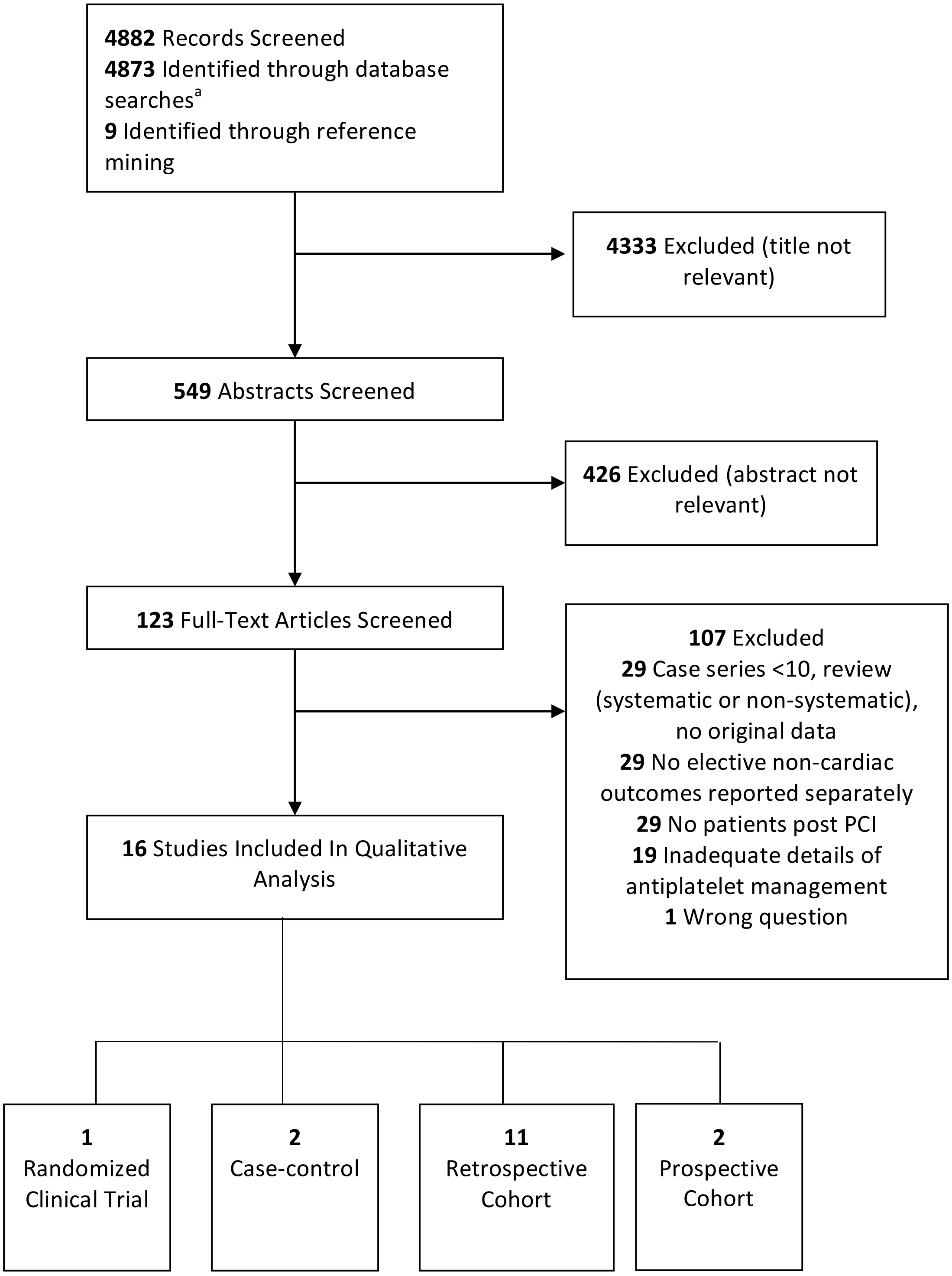

PCI = percutaneous coronary intervention

a. Search results were combined from Scopus, Web of Science, and PubMed. See Appendix 2 for details.
